# Sleep environment is associated with sleep control in fly-in, fly-out mining shift workers

**DOI:** 10.1007/s11325-025-03454-5

**Published:** 2025-09-18

**Authors:** Philipp Beranek, Mitchell Turner, Johnny Lo, Michael Grandner, Ian C. Dunican, Travis Cruickshank

**Affiliations:** 1 School of Medical and Health Sciences, Edith Cowan University, Joondalup, WA Australia; 2 Centre for Precision Health, Edith Cowan University, Joondalup, WA Australia; 3 Perron Institute for Neurological and Translational Sciences, Perth, WA Australia; 4 Melius Consulting, North Fremantle, WA Australia; 5 Sleep and Health Research Program, Department of Psychiatry, University of Arizona, Tucson, AZ USA; 6 UAHS Center for Sleep and Circadian Sciences, University of Arizona, Tucson, AZ USA; 7 School of Science, Edith Cowan University, Perth, WA Australia

**Keywords:** Sleep timing, FIFO, Sleep quality, Camp, Accommodation, Sleep hygiene

## Abstract

Control over sleep timing, duration, and quality is essential for obtaining healthy sleep. Fly-in, Fly-out (FIFO) mining shift workers face unique challenges in maintaining control over their sleep due to their roster. However, the impact of their sleep environment on perceived sleep control in this population remains underexplored. This study evaluates the sleep environment of FIFO mining shift workers and its association with sleep control. A cross-sectional study was conducted (2023–2024) on Australian FIFO mining shift workers residing in remote camp accommodations. Data were collected via an online survey using the Assessment of Sleep Environment (ASE) and the Brief Index of Sleep Control (BRISC). Linear regression and Random Forest analyses were used to explore associations and identify key environmental factors influencing sleep control. Respondents (*n* = 538, 29% female) reported a mean ASE score of 12.6 ± 7.4 and a BRISC score of 2.3 ± 0.9. Two-thirds (*n* = 366) rated their sleep environment as moderately or highly sleep-disruptive. Higher ASE scores were negatively associated with BRISC scores (*p* < 0.001). Key environmental factors included pillow/blanket comfort, mattress comfort, room temperature, noise, light, and safety, of which all were negatively correlated (*p* < 0.05) with sleep control. The findings highlight the importance of optimising sleep environments to improve FIFO mining shift workers’ sleep control. Addressing bedding comfort, noise and light levels, temperature, and safety could improve sleep in this population. However, the cross-sectional design does not allow for the identification of causal relationships. Longitudinal and objective studies are warranted to confirm causality and guide interventions.

**Supplementary Information:**

The online version contains supplementary material available at 10.1007/s11325-025-03454-5.

## Introduction

Sleep health is vital for everyday functioning and overall health [1]. Shift work is known to impair sleep, increasing the risk of fatigue-related consequences (e.g., accidents) and adverse long-term health outcomes (e.g., cardiovascular disease) [2, 3]. Shift workers face significant challenges in controlling their sleep timing, duration, and quality due to limited autonomy over their roster [4–6]. Studies on mining shift workers consistently report an average of less than 7 h of sleep duration and poor sleep quality [7–9]. This reduction in duration and quality may be attributed to roster-related factors, including shift duration (~ 12 h), shift type (e.g., night shift), roster type (e.g., rotating shifts), shift start times (e.g., ≤ 06:00) and number of consecutive shifts (e.g., 14) [7–9]. Whilst improving roster design may increase control over sleep [8], the feasibility is limited given the industry’s reliance on 24/7 operations. Therefore, modifiable factors potentially influencing control over sleep, such as the sleep environment, warrant investigation.

Environmental factors, including temperature, light, air quality, bedding and noise, can impact sleep [10, 11]. Research in the general population has shown a positive relationship between sleep environment satisfaction and sleep quality, with key environmental factors including ambient temperature, noise level, humidity, light, and air quality [12]. Delayed time at sleep onset, increased awakenings, and altered sleep architecture have also been reported when individuals are exposed to poor sleep environment conditions (e.g., light level ≥ 5 lx), highlighting the importance of a sleep-conducive bedroom set-up [10, 13].

In Australia, mining sites are often located in remote regions such as the Pilbara, with a long distance (e.g., 1500 km) to the nearest major city (e.g., Perth). This remoteness necessitates a fly-in fly-out (FIFO) arrangement, where employees fly to the mining sites (fly-in) and complete consecutive ~ 12-hour shifts (day or night) over extended periods (e.g., two weeks) before flying home (fly-out) for time off work (e.g., two weeks) [14]. Between shifts, FIFO mining shift workers stay and sleep in mining camps. Purpose-built accommodations often consist of transportable containers with basic amenities such as a single bed, fridge, desk, ensuite bathroom, and air conditioning (Supplementary items S1 and S2). Despite evidence that 53% of FIFO mining shift workers report dissatisfaction with their accommodations [15], limited research has focused on the impact of sleep environment factors on their ability to initiate and maintain sleep. For instance, daylight entering the room and an elevated room temperature due to excessive outdoor temperatures (> 40 °C) may disrupt the sleep of night shift workers. Further, thin walls of the rooms make the sleep environment vulnerable to outside noise (e.g., cars and neighbours).

To the best of our knowledge, no previous study has investigated the relationship between the sleep environment and sleep control in FIFO mining shift workers residing in mining camps. For the first time, this study evaluates the sleep environment of FIFO mining shift workers and the extent to which they feel in control over their sleep. Associations between the sleep environment and perceived control over sleep are investigated. Based on existing literature, we hypothesised that FIFO mining shift workers are exposed to environmental factors that disrupt their sleep and experience reduced control over their sleep patterns. Furthermore, we hypothesised that a more conducive sleep environment would be associated with greater sleep control. Finally, we hypothesised that individual sleep environment factors would influence perceived sleep control differently among FIFO mining shift workers.

## Methods and materials

### Study design

This study employed a cross-sectional design to investigate FIFO mining shift workers’ sleep environment and their control over sleep. The data presented is from a research program that examines sleep in shift workers in extractive industries in Australia.

An online survey was administered via REDCap (Research Electronic Data Capture software) between June 2023 and February 2024. The FIFO mining shift workers were recruited through online, print, and in-person strategies, including social media, magazines, conferences and airport-based outreach. A website (www.sleeproom.au) was established to provide relevant study information. Participants provided informed consent (tick box) prior to their commencement of the study. The Edith Cowan University Human Research Ethics Committee (2023–04157) provided ethical approval for this study.

### Participants

Participants in this study were adults in Australia who worked in fly-in-fly-out (FIFO) mining as shift workers and slept in mining camp accommodations between consecutive shifts. Inclusion criteria were as follows: (1) aged ≥ 18 years, (2) working as a shift worker, (3) working in the mining industry, (4) having an Australian residential address, and (5) sleeping away from home when undertaking shifts.

### Measures

#### Demographic information

Participants completed demographic questions on age, sex, height, weight, education level, combined annual household income, and relationship status. Several work-related questions were also administered, capturing information on the type of employment (e.g., Full-time, part-time, casual), years of shift work experience, and type of shifts (e.g., Permanent day shift, forwards rotating shifts).

#### Sleep environment assessment

The Assessment of Sleep Environment (ASE) questionnaire was administered to determine the perceived level of disruptiveness of sleep environmental factors on sleep. The ASE consists of 13 items, assessing light, noise, temperature, humidity, smell, comfort, and safety. Participants rate each item on a 4-point Likert scale. Scoring for each item is from 0 (strongly disagree) to 3 (strongly agree). A total score is created by summing the individual values, ranging from 0 to 39. The perceived level of sleep disruptiveness is interpreted as follows: A score of 0 to 9 is considered low, 10 to 19 is considered moderate, and 20 to 39 is considered high [16]. The ASE has demonstrated adequate validity with the Insomnia Severity Index (ISI) and the Pittsburgh Sleep Quality Index (PSQI) [16].

#### Brief index of sleep control

Control over their sleep was assessed using the Brief Index of Sleep Control (BRISC). The BRISC was developed to evaluate the degree to which participants feel they have control over their sleep, including when they go to sleep and wake up and how much and how well they sleep [4]. The BRISC consists of 4 items. Participants are asked to rate each item on a 5-point Likert Scale. Each item is scored from 0 (none) to 4 (complete control). A score is created by calculating the mean of all items, ranging between 0 and 4, with a higher score indicating more control over sleep [4]. There is a significant correlation between the BRISC and the PSQI, ISI, Epworth Sleepiness Scale (ESS), and total sleep time, demonstrating its validity as a measure of sleep control [4].

### Statistical analysis

Data is presented as mean ± Standard Deviation or frequency (%) and count (n). Univariate and multivariate linear regression models were performed to address the primary aim of determining the relationships between control over sleep (BRISC) and age, sex, shift type, years of shift work experience, and sleep environment (ASE). A T-test was performed to determine sex differences in ASE scores. The Chi^2^ test was used to determine response distribution differences in individual ASE items and individual BRISC items between males and females. An exploratory analysis using a random forest was used to determine the importance of individual environmental factors (individual ASE items) on control over their sleep (BRISC). The increased node purity (IncNodePurity) score was used to assess variable importance. As post-hoc analysis, Spearman’s Rank Correlation analyses were performed to assess the direction of the relationship between the most important environmental factors (individual ASE items) and control over sleep (BRISC). False discovery rate (FDR) corrections were applied to account for multiple comparisons and mitigate false-positive results. A statistical significance level of *p* < 0.05 was defined. Using G*Power, the minimum required sample size to detect a medium effect size (f² = 0.15) at 80% power and a 5% level of significance is 118. The conventional Cohen’s medium effect size was chosen, given that no similar studies were available on the BRISC in this population. Statistical analyses were performed using R Studio (Version 2024.04.02) and Python software (Version 3).

## Results

### Participants information

A total of 602 FIFO mining shift workers commenced the survey, with 89% (*n* = 538) completing it. Of the respondents (*n* = 538), 71% (*n* = 384) were male, and 29% (*n* = 154) were female. Demographic and sociodemographic information is reported in Table 1.

**Table 1:** Demographic and sociodemographic information

	Values	
Age (years, mean ± SD)	39 ± 12	
Height (cm)	175 ± 10	
Weight (kg)	85 ± 18	
BMI (mean ± SD)	28 ± 5	
Sex		
Male	71%	(*n* = 384)
Female	29%	(*n* = 154)
Education level		
High school or less	19%	(*n* = 101)
Diploma, certificate/trade	47%	(*n* = 251)
Bachelor’s degree	25%	(*n* = 136)
Master’s degree	9%	(*n* = 48)
Doctoral degree	< 1%	(*n* = 2)
Relationship status		
Married	38%	(*n* = 202)
Single	29%	(*n* = 157)
Unmarried, in a relationship, living together	20%	(*n* = 108)
Unmarried, in a relationship, not living together	8%	(*n* = 44)
Divorced or legally separated	4%	(*n* = 24)
Widowed	< 1%	(*n* = 3)
Combined annual household income (AUD)		
Less than $80,000	5%	(*n* = 28)
$80,000 to $150,000	29%	(*n* = 158)
$150,001 to $300,000	55%	(*n* = 298)
$300,001 to $500,000	9%	(*n* = 48)
$500,001 to $750,000	< 1%	(*n* = 4)
More than $750,000	< 1%	(*n* = 2)
Employment level		
Casual	12%	(*n* = 63)
Part-time	1%	(*n* = 6)
Full-time	87%	(*n* = 469)
Shift type		
Fixed shifts	51%	(*n* = 272)
Rotating shifts	31%	(*n* = 168)
Variable shifts (ad hoc)	18%	(*n* = 98)
Shift work experience		
Less than 5 years	45%	(*n* = 242)
5 to 10 years	20%	(*n* = 109)
10 to 15 years	16%	(*n* = 87)
15 to 20 years	10%	(*n* = 53)
More than 20 years	9%	(*n* = 47)

Data presented as frequency (percentage, %) and count (n) unless stated otherwise. Percentages have been rounded, and < 1% has been used for unrounded percentages smaller than 1%

### Sleep environment

The mean ASE score was 12.6 ± 7.4, with a range of 0.0 to 39.0. More than half of the respondents (55%, *n* = 294) perceived the sleep-disruption level of their sleep environment as moderate, followed by low (32%, *n* = 172) and high (13%, *n* = 72). While there was no significant (*p* = 0.21) sex difference for the ASE scores, there was a significant (*p* = 0.01) response distribution difference between males and females in one environmental factor: not feeling safe and secure. The response distribution for individual ASE items is reported in (Supplementary item S3).

### Control over sleep

**Fig. 1:**
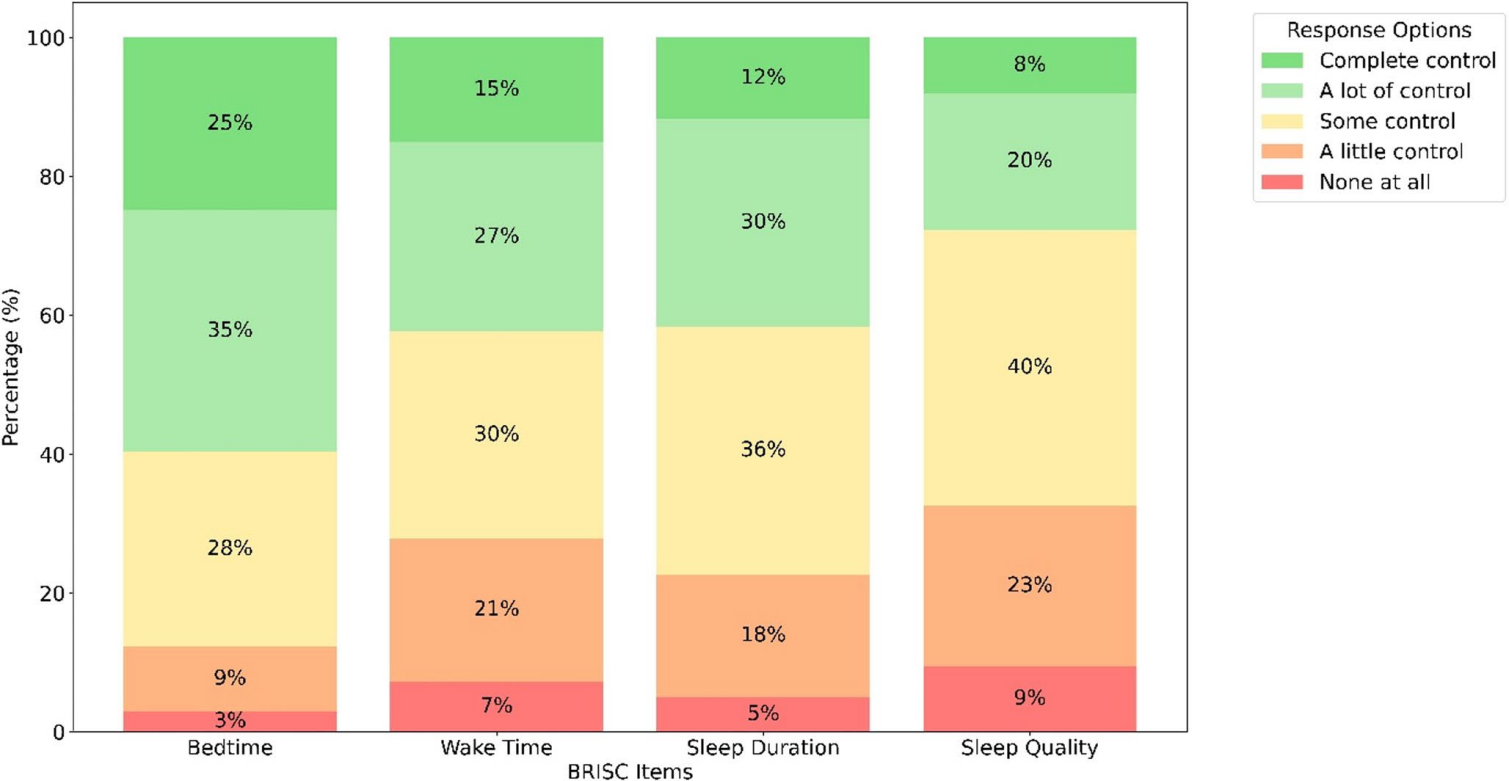
Response distribution for individual BRISC items

The mean BRISC score was 2.3 (± 0.9), with a range of 0.0 to 4.0. There was no significant difference between males and females in the BRISC scores (*p* = 0.45) and response distribution in individual item scores (*p* > 0.05 for all). The response distribution for individual BRISC items is presented in Fig. 1.

### Associations

**Fig. 2:**
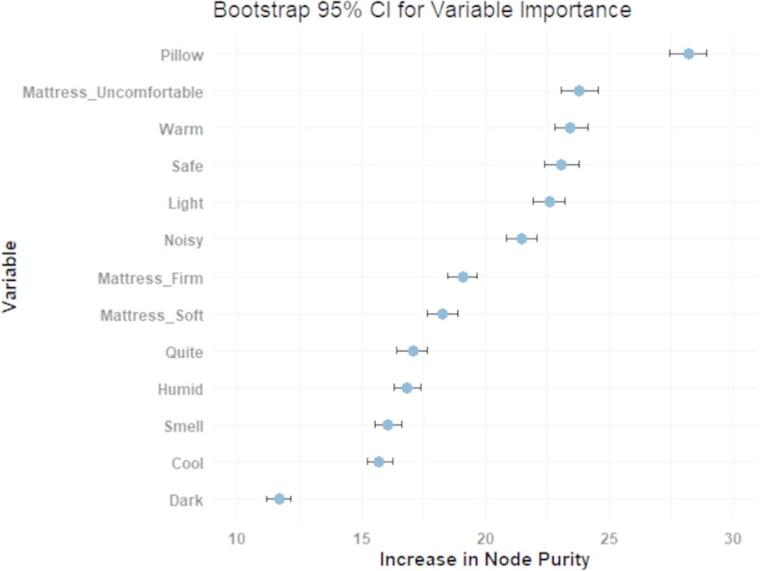
Variable importance of ASE items in predicting the BRISC score using Random Forest. The error bars represent 95% bootstrap confidence intervals (CIs), calculated from 1,000 bootstrap samples. The stability and reliability of the variable importance measures are reflected in the narrow widths of the CIs

The ASE scores were negatively associated with the BRISC scores (*p* < 0.001). Specifically, those with low ASE scores had higher BRISC scores (2.54 ± 0.06) compared to those with moderate (2.19 ± 0.05, *p* < 0.001) or high (2.03 ± 0.10, *p* < 0.001) ASE scores. An increase in age was significantly associated with a lower ASE score (*p* = 0.03) and a higher BRISC score (*p* < 0.001). These associations remained significant upon FDR correction (*p* < 0.05 for all). The random forest model identified six ASE items of high importance for control over sleep (see Fig. 2). Pillow/blanket comfort (IncNodePurity: 28.2) was identified as the most important factor for control over sleep, followed by five ASE items related to mattress comfort (IncNodePurity: 23.8), temperature (IncNodePurity: 23.4), safety (IncNodePurity: 23.1), light (IncNodePurity: 22.6), and noise (IncNodePurity: 21.5). All six factors were negatively correlated with BRISC scores (*p* < 0.05 for all). These correlations remained significant upon FDR correction (*p* < 0.05 for all).

## Discussion

This study investigated, for the first time, the sleep environment and sleep control and their respective association in FIFO mining shift workers. Consistent with our hypotheses, two-thirds of the FIFO mining shift workers perceived their sleep environment as moderately or highly sleep-disruptive. Approximately every tenth FIFO mining shift worker reported little to no control over bedtime, every fourth reported little to no control over waketime, every fourth reported little to no control over sleep duration, and approximately every third reported little to no control over sleep quality. The FIFO mining shift workers who experienced a more sleep-conducive environment reported greater sleep control. Individual environmental factors related to bedding comfort, room temperature, light, noise level, and sense of safety contributed the most to sleep control in FIFO mining shift workers.

### Sleep environment perception of mining shift workers

We found that FIFO mining shift workers experienced a moderately sleep-disturbing environment (average ASE: 12.6) in mining camps. This finding was unexpected, given that mining camp accommodations typically consist of small, interconnected transportable units often situated in extreme climates (e.g., heat), with limited adjustments possible, making them susceptible to environmental disturbances such as noise. In our study, a considerable percentage of our sample reported a moderate to highly disruptive sleep environment. This finding aligns with previous research in offshore workers, who reported experiencing disruptive environmental factors [17]. This suggests that mining companies may want to consider adjustments to existing mining camps to improve sleep conditions in these settings.

### Sleep control of mining shift workers

We observed an average BRISC score of 2.3 in FIFO mining shift workers. This finding was surprising given that poor sleep outcomes, such as poor sleep quality and short sleep duration, have been widely reported in FIFO mining shift workers and associated with their roster [7, 8]. Given that a large proportion of our sample reported having little or no control over sleep quality and sleep duration, aligning with research in similar industries [18], resources should be provided to FIFO mining shift workers on how to obtain more sleep and achieve better sleep quality when sleeping in mining camps. This may include education on sleep hygiene practices, such as setting the room temperature to between 16 and 20 °C and following a bedtime routine [19]. At the organisational level, further avenues for improving FIFO mining shift workers’ sleep control, such as adjusting roster designs, are of interest.

###  Associations between sleep environment and sleep control

Our study found that FIFO mining shift workers who sleep in a more sleep-conducive environment experienced greater control over their sleep. This is consistent with previous work, which found that poor sleep environments (e.g., high room temperature and disruptive noise) negatively impact sleep outcomes, including sleep onset latency and wake after sleep onset [10], indicating a detrimental impact on sleep control. In offshore workers, higher noise levels, vibrations, and poor air quality were associated with poorer sleep quality and trouble sleeping [17]. The relationship between sleep environment and sleep control may be bidirectional. Individuals with low control over their sleep may be experiencing difficulties falling and staying asleep, thus being more likely to perceive sleep-disturbing environmental factors (e.g., outside noise, safety concerns). This is supported by findings in people living with insomnia, where less sleep-conducive environments have been found to be associated with greater insomnia symptom severity [16]. There is a need to better understand and address environmental factors that negatively impact FIFO mining shift workers’ sleep control.

### Sleep environment factors and sleep control

Six environmental factors contributed the most to sleep control in the random forest model. These factors are related to bedding comfort, room temperature, light exposure, noise levels and safety perception.

#### Bedding comfort

To date, limited research has examined the influence of bedding on sleep. Existing studies have reported increased waking symptoms and reduced sleep quality when individuals sleep on uncomfortable pillows or mattresses [20, 21]. In a qualitative investigation conducted across various industries, participants highlighted the importance of having a comfortable bed in work accommodations [18]. This aligns with our findings, where experiencing an uncomfortable pillow/blanket and mattress discomfort were correlated with lower sleep control. Several bedding characteristics, including firmness, thickness and material, are known to influence sleep [22]. Given the affordability and feasibility of improving bedding comfort, mining companies may consider offering various pillow and mattress options to suit individual preferences [23].

#### Room temperature

In our study, a too warm sleep environment was correlated with lower sleep control. Reduced self-reported sleep quality has been previously reported in individuals who perceive bedroom temperature as too warm [12]. Offshore workers frequently reported that cabin temperature causes sleep problems [24]. Excessive room temperatures increase core body temperature and contribute to delayed sleep onset and nocturnal awakenings [25].

#### Light exposure

Evening and night-time light exposure have been shown to disrupt sleep by delaying sleep onset and increasing nighttime awakenings [26, 27]. Consistent with previous work, too much light in the sleep environment was correlated with lower sleep control. Light exposure in the evening delays sleep onset and nocturnal light exposure increases nocturnal awakenings [28, 29]. Studies mitigating nighttime light exposure have reported positive effects on sleep, including reduced sleep onset latency and wake after sleep onset [30, 31]. Thus, mining companies may want to consider alternative lighting that allows setting low light levels in camp accommodations, whilst shift workers may want to cover light-emitting sources (e.g., light from electronic devices).

#### Noise levels

We found that having a too noisy sleep environment was correlated with lower sleep control in FIFO mining shift workers, which may be due to the thin walls of mining camp accommodations. Similar findings were reported in offshore workers. In that study, noise was associated with sleep quality, with reported sources such as noise from ventilation and power generation [17]. Given the potential impact of noise on FIFO mining shift workers’ sleep, mining companies should consider implementing noise-reduction strategies, such as soundproofing accommodations. Additionally, identifying sources of sleep-disruptive noise would allow to reduce noise levels in mining camps.

#### Safety perception

There is limited research on the relationship between safety perception and sleep. Initial research observed a link between neighbourhood crime and poor sleep [32], suggesting that poor sleep may be linked to low safety perception. A study on patients’ sleep in the healthcare setting found that patients felt unsafe because they were unsure of what was happening on the ward [33]. This aligns with our finding, where feeling less safe and secure was correlated with lower sleep control in FIFO mining shift workers. Feeling unsafe increases the level of arousal, negatively affecting sleep [34]. This may be relevant when shift workers sleep on site, where they may be alone in the room, and limited safety measures (e.g., safety screens and alarm systems) may be available compared to at home. Mining companies may want to consider implementing additional safety measures (e.g., security personnel) to increase safety perception while asleep.

### Limitations

This study addressed key gaps in the literature but has several limitations. Its cross-sectional design precludes the examination of longitudinal relationships. Therefore, it is essential to note that the cross-sectional design employed in this study does not permit the identification of causal relationships, and the findings presented herein should be interpreted with caution. Additionally, reverse causality, where poor sleep control influences perceptions of the sleep environment, is also possible and cannot be ruled out in this study. Consequently, it is possible that respondents experiencing poor sleep control were more likely to perceive their sleep environment as disruptive. However, this approach was deemed appropriate given the high staff turnover in the mining industry and the limited existing evidence in this area. Future studies are encouraged to use longitudinal or experimental study designs. Additionally, self-report measures were used, which, while allowing for data collection from a large sample (*n* = 538), are subject to sample bias, recall bias, and estimation error. Future research should incorporate objective measures [7], such as actigraphy to assess sleep-wake behaviour or home-based polysomnography to evaluate sleep architecture, to enhance data accuracy. Our recruitment strategy involved open invitations through various public channels. Therefore, the total number of individuals who viewed or were exposed to the study invitation is unknown and it is not possible to derive the response rate. Given the voluntary nature of participation, self-selection bias is possible. Individuals with a greater interest in or concern about sleep may have been more inclined to participate, potentially limiting the generalisability of the findings to all FIFO workers. Although a significant relationship was found between individual sleep environment factors and control over sleep, the direction and underlying mechanisms remain unclear.

## Conclusions

This is the first study investigating the relationship between the sleep environment and control over sleep in FIFO mining shift workers. The demonstrated relationship between the sleep environment and control over sleep herein indicates that the sleep environment may be a crucial factor in FIFO mining. Mining companies may, therefore, want to assess and improve existing accommodations. Bedding comfort, room temperature, light and noise levels, and security may be environmental factors to consider. However, longitudinal research is warranted to investigate the cause-effect relationship between the above sleep environment factors and sleep control in FIFO mining shift workers.

## Supplementary Information

Below is the link to the electronic supplementary material.

Supplementary Material 1

## Data Availability

Data will be made available upon reasonable request.
