# Supplementary material for: Sleep environment is associated with sleep control in fly-in, fly-out mining shift workers
Source: Sleep Breath. 2025 Sep 18;29(5):289. doi: 10.1007/s11325-025-03454-5 (PMC12446090; doi:10.1007/s11325-025-03454-5)
Supplement: Supplementary file 1 — Supplementary Material 1 [file 11325_2025_3454_MOESM1_ESM.docx]

Journal: Sleep and Breathing

**Sleep environment is associated with sleep control in fly-in, fly-out mining shift workers**

Philipp Beranek, MSc^1,2^, Mitchell Turner, PhD^1,2^, Johnny Lo, PhD^7^, Michael Grandner, PhD^5,6^, Ian C. Dunican, PhD^,4^, Travis Cruickshank, PhD^1,2,3^

**Affiliations:**

1 School of Medical and Health Sciences, Edith Cowan University, Joondalup, Western Australia, Australia

2 Centre for Precision Health, Edith Cowan University, Joondalup, Western Australia, Australia

3 Perron Institute for Neurological and Translational Sciences, Perth, Western Australia, Australia

4 Melius Consulting, North Fremantle, WA, Australia

5 Sleep and Health Research Program, Department of Psychiatry, University of Arizona, Tucson, AZ

6 UAHS Center for Sleep and Circadian Sciences, University of Arizona, Tucson, AZ

7 School of Science, Edith Cowan University, Perth, WA, Australia

**Corresponding Author:**

Mr Philipp Beranek, MSc

Email: p.beranek@ecu.edu.au


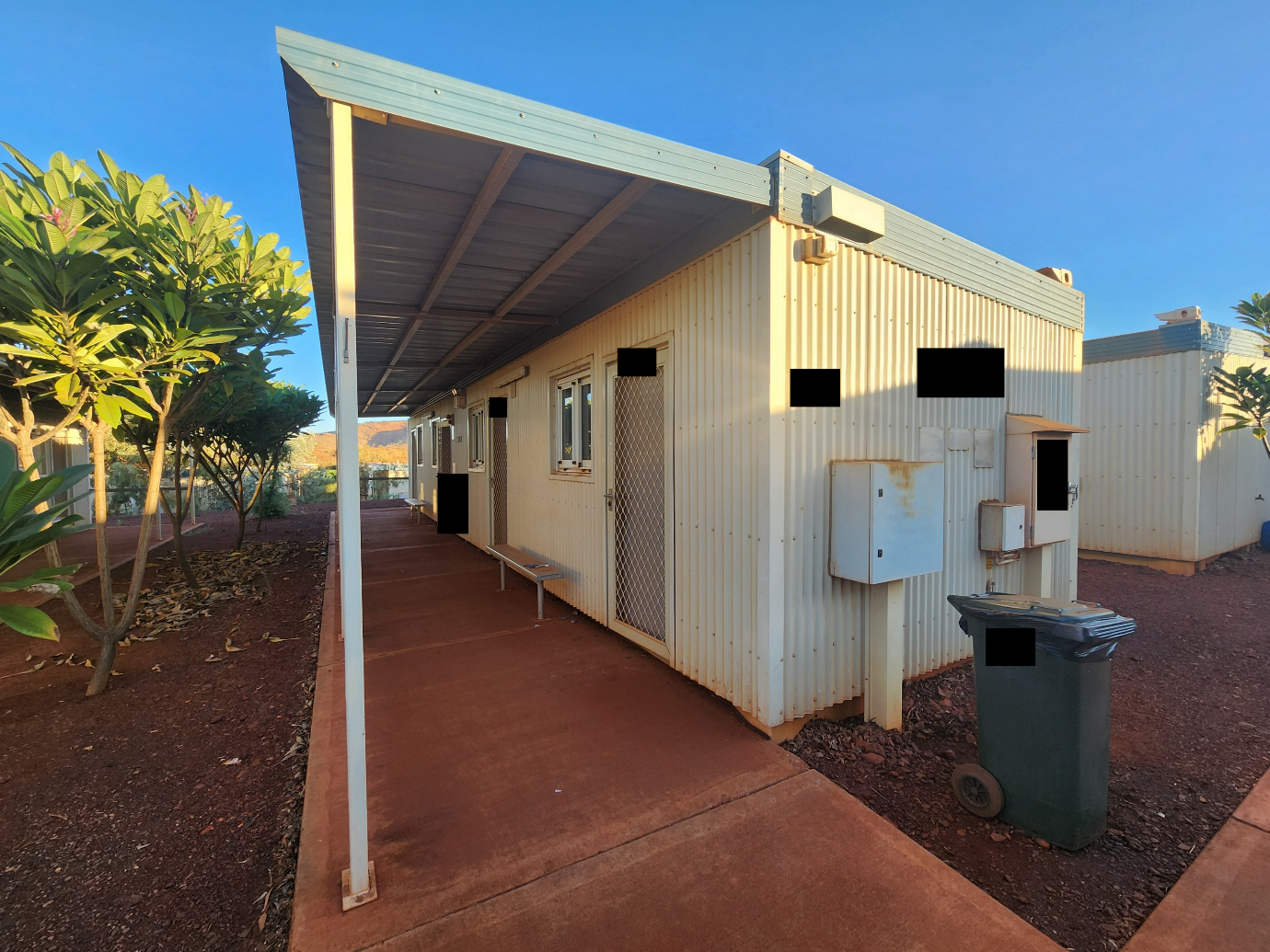


***Supplementary item S1*** Mining camp accommodation from outside


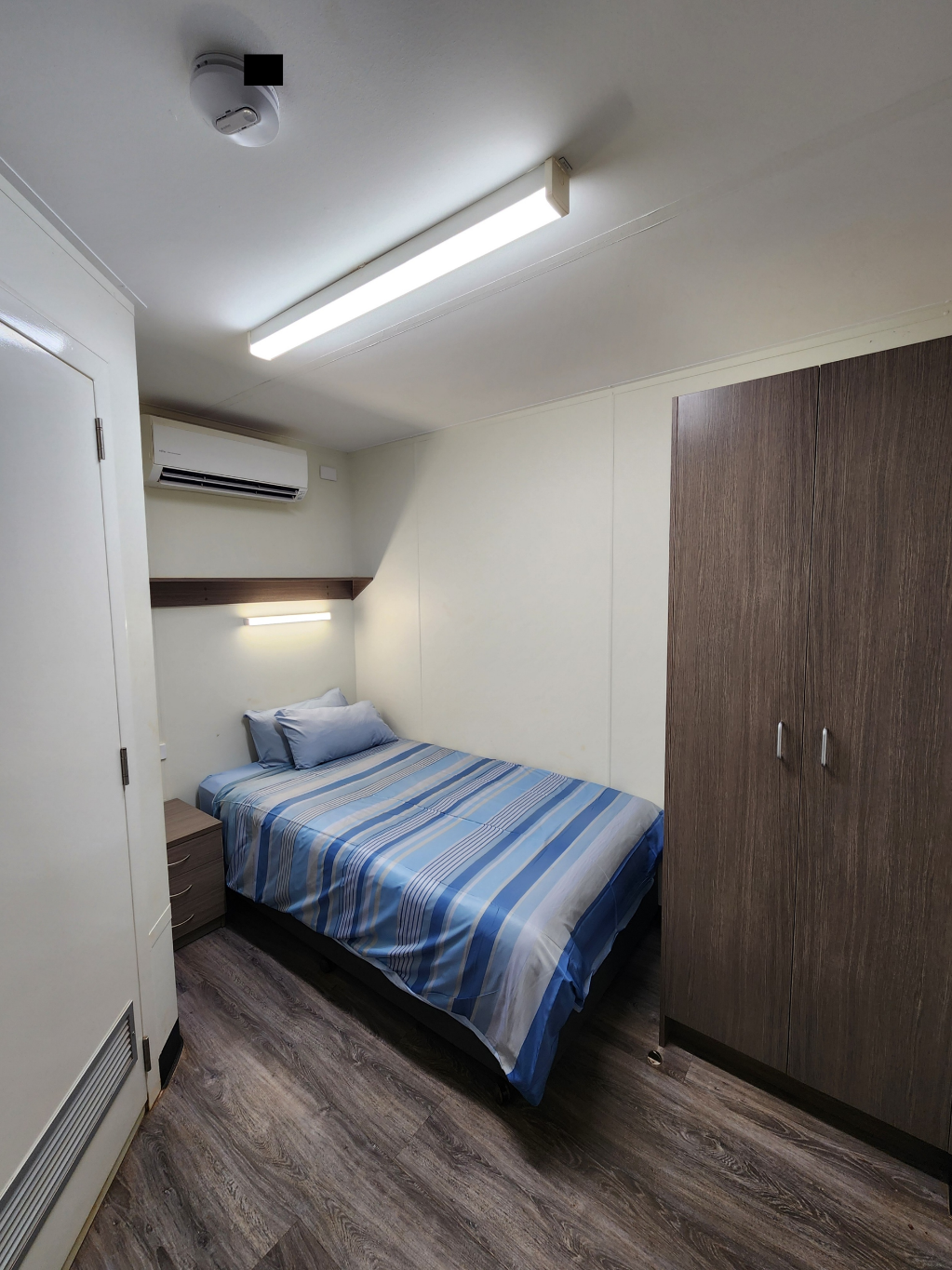


***Supplementary item S2*** Mining camp accommodation from inside

| ***Supplementary item S3*** Response distribution for individual ASE items | | |
| --- | --- | --- |
|  |  | |
|  | **Frequency** | |
| **Too much light** | | |
| Strongly Disagree | 32% | (n=173) |
| Disagree | 43% | (n=230) |
| Agree | 16% | (n=87) |
| Strongly Agree | 9% | (n=48) |
| **Too dark** | | |
| Strongly Disagree | 60% | (n=323) |
| Disagree | 37% | (n=201) |
| Agree | 1% | (n=6) |
| Strongly Agree | 1% | (n=8) |
| **Too noisy** | | |
| Strongly Disagree | 29% | (n=157) |
| Disagree | 39% | (n=208) |
| Agree | 23% | (n=124) |
| Strongly Agree | 9% | (n=49) |
| **Too quiet** | | |
| Strongly Disagree | 54% | (n=291) |
| Disagree | 39% | (n=211) |
| Agree | 5% | (n=27) |
| Strongly Agree | 2% | (n=9) |
| **Too warm** | | |
| Strongly Disagree | 24% | (n=128) |
| Disagree | 38% | (n=203) |
| Agree | 29% | (n=157) |
| Strongly Agree | 9% | (n=50) |
| **Too cool** | | |
| Strongly Disagree | 36% | (n=195) |
| Disagree | 51% | (n=273) |
| Agree | 11% | (n=58) |
| Strongly Agree | 2% | (n=12) |
| **Too humid** | | |
| Strongly Disagree | 34% | (n=182) |
| Disagree | 43% | (n=234) |
| Agree | 15% | (n=83) |
| Strongly Agree | 7% | (n=39) |
| **Uncomfortable smell** | | |
| Strongly Disagree | 37% | (n=198) |
| Disagree | 39% | (n=209) |
| Agree | 16% | (n=88) |
| Strongly Agree | 8% | (n=43) |
| **Uncomfortable pillows or blankets** | | |
| Strongly Disagree | 18% | (n=96) |
| Disagree | 27% | (n=144) |
| Agree | 36% | (n=194) |
| Strongly Agree | 19% | (n=104) |
| **Mattress/other surface too firm** | | |
| Strongly Disagree | 25% | (n=134) |
| Disagree | 39% | (n=212) |
| Agree | 23% | (n=124) |
| Strongly Agree | 13% | (n=68) |
| **Mattress/other surface too soft** | | |
| Strongly Disagree | 34% | (n=184) |
| Disagree | 47% | (n=254) |
| Agree | 14% | (n=75) |
| Strongly Agree | 5% | (n=25) |
| **Mattress/other surface uncomfortable for other reason** | | |
| Strongly Disagree | 29% | (n=155) |
| Disagree | 37% | (n=198) |
| Agree | 23% | (n=124) |
| Strongly Agree | 11% | (n=61) |
| **Not safe and secure** | | |
| Strongly Disagree | 47% | (n=252) |
| Disagree | 41% | (n=219) |
| Agree | 9% | (n=48) |
| Strongly Agree | 4% | (n=19) |
| **Note:** Data presented as frequency (percentage, %) and count (n). Percentages have been rounded. | | |

**Practical Sleep Environment Modifications for Remote Mining Camps**

**Bedding Comfort**

**Management:**

- *Pillow:* Offering different types of pillows may allow individuals to customise their bedding and improve comfort.
- *Mattress:* Where possible, mining camp providers should consider offering a range of mattress types to accommodate individual preferences. All mattresses should be regularly inspected and replaced as needed.
- *Bedding:* Bedding, such as bed sheets, should be changed regularly to maintain hygiene.

**Individuals:**

- *Pillow:* Bringing a comfortable pillow to the mining camp can improve sleep quality. It can be kept at the accommodation in a lockable cupboard.
- *Mattress:* If the provided mattress is uncomfortable and cannot be exchanged, a mattress topper can offer additional support and comfort. Any concerns regarding mattress hygiene should be reported to the camp management.
- *Bedding:* If camp-provided sheets are uncomfortable, using personal bed linen may enhance sleep comfort. Concerns regarding bedding hygiene or discomfort should be reported to the camp management.

**Room** **Temperature**

**Management:**

- Mining camp providers should ensure appropriate air conditioning units are installed, allowing sufficient cooling on hot days (e.g., >40°C).
- Adding heat insulation to the accommodation structure can further support thermal comfort.
- Providing several blankets can help adjust thermal comfort in bed.

**Individuals:**

- Setting a cool temperature in your room (e.g., 16 to 20°C) can facilitate falling asleep and reduce awakenings.
- Issues with air conditioning units should be reported to camp management.
- Use of a personal blanket may help with body temperature regulation.

**Light Exposure**

**Management:**

- Installing adjustable lighting systems allows control over brightness levels.
- Installing blackout blinds avoids outside light from entering the room through windows.
- It should be ensured that any gaps (e.g., around the door) are sealed.

**Individuals:**

- Light exposure before bedtime should be reduced. This can be achieved by using only minimal light sources installed or personal lamps.
- To block outdoor light, blackout blinds should be used. If blinds are improperly fitted or ineffective, workers should report the issue to camp management. Windows can also be sealed using temporary materials (e.g., tape) to reduce light intrusion.
- Lights from appliances (e.g., air conditioner) that disturb sleep can be covered with tape, if appropriate, or discussed with camp management.
- Wearing an eye mask can be beneficial when light in the room cannot be avoided.

**Noise**

**Management:**

- Assigning neighbouring rooms to workers on opposite shifts should be avoided. Assigning blocks or camp areas to workers on the same shift (e.g., night shift) can help reduce disruptive noise through cleaning or maintenance work in adjacent rooms.
- Camp designs should consider the car park locations and design to avoid disruptive noise in nearby rooms.
- Acoustic fencing may reduce noise transmission through the camp.

**Individuals:**

- Placing the “Night Shift” sign on the outside of the door alerts camp staff and colleagues.
- If neighbouring noise is disruptive, politely discussing the issue with the neighbour may resolve the problem.
- Noisy appliances (e.g., rattling air conditioner) should be reported for maintenance.
- Where external noise cannot be eliminated, using earplugs or white noise apps may help mask disturbances.

**Safety Perception**

**Management:**

- Measures that may improve the sense of safety include
  - increased security personnel presence
  - Installation of safety screens
  - improved locks

**Individuals:**

- If a worker feels unsafe in their accommodation, this should be addressed with the camp management.
